# Spesolimab in the Management of Generalized Pustular Psoriasis With Concurrent Bullous Pemphigoid and Psoriasis

**DOI:** 10.7759/cureus.60331

**Published:** 2024-05-15

**Authors:** Romane Teshima, Natsuko Saito-Sasaki, Taiyo Hitaka, Yu Sawada

**Affiliations:** 1 Dermatology, University of Occupational and Environmental Health, Kitakyushu, JPN

**Keywords:** treatment, dermatology case report, il-36, bullous pemphigoid, generalized pustular psoriasis (gpp)

## Abstract

Autoimmune diseases often co-occur due to shared immunological mechanisms, necessitating strategic treatment approaches to manage overlapping conditions without exacerbating each other. A 75-year-old male with a history of psoriasis vulgaris and bullous pemphigoid (BP) developed new-onset pustular psoriasis under systemic corticosteroid therapy, which is known to potentially worsen psoriasis into its pustular form. Histological examination confirmed the diagnosis, showing features typical of pustular psoriasis. The patient was successfully treated with spesolimab, an anti-IL-36 neutralizing antibody, achieving complete remission without aggravating the BP. This case highlights the necessity of cautious treatment selection in patients with multiple autoimmune disorders and underscores the potential role of IL-36 in exacerbating inflammatory responses in BP. Further research into the interaction between IL-36 and BP may provide deeper insights into managing such complex clinical scenarios.

## Introduction

Autoimmune diseases often occur simultaneously due to overlapping immune system dysfunctions [1-3]. Understanding these shared mechanisms is crucial for devising treatment strategies that effectively manage one condition without exacerbating another [4]. In the clinical context, managing concurrent autoimmune diseases like bullous pemphigoid (BP) and psoriasis presents unique challenges [5]. Specifically, the use of systemic corticosteroids for treating BP can inadvertently trigger exacerbations of pustular psoriasis [6]. This interplay complicates treatment decisions, particularly because the coexistence of BP and psoriasis is rare, thus limiting the availability of well-established treatment protocols. Such complexities make the clinical management of these cases particularly challenging. In this report, we describe a successful intervention using spesolimab in a patient with both pustular psoriasis and BP, highlighting a potential therapeutic approach in similar complex clinical scenarios.

## Case presentation

**Figure 1:**
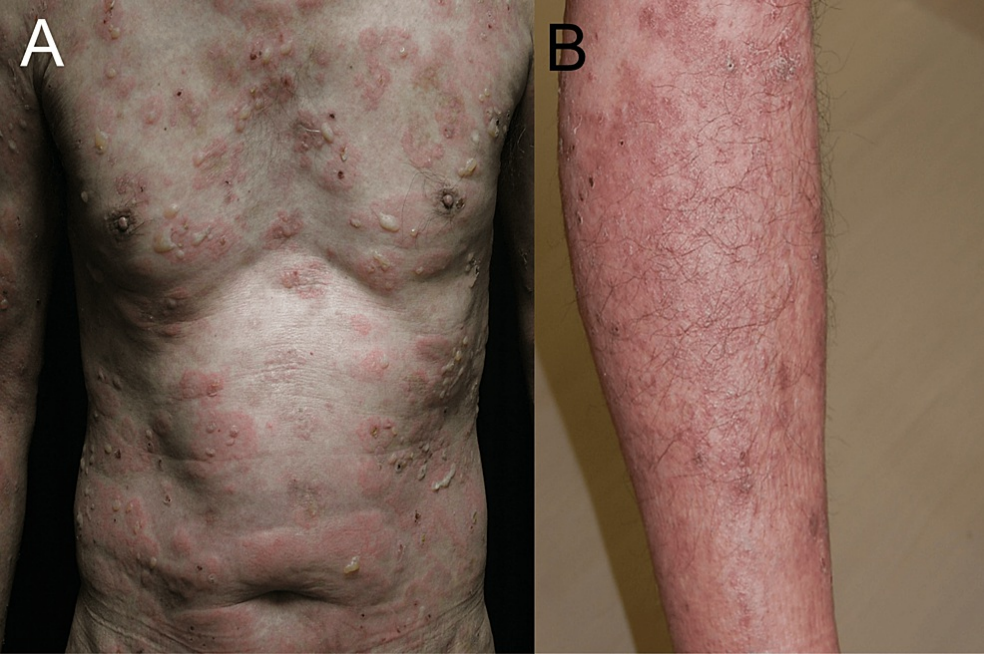
Clinical manifestation of BP and psoriasis (A, B) Clinical manifestations of concurrent BP and psoriasis. (A) Tense blisters were distributed over the erythematous plaques. (B) Scaly erythematous plaques were located on the lower legs. BP, bullous pemphigoid

**Figure 2:**
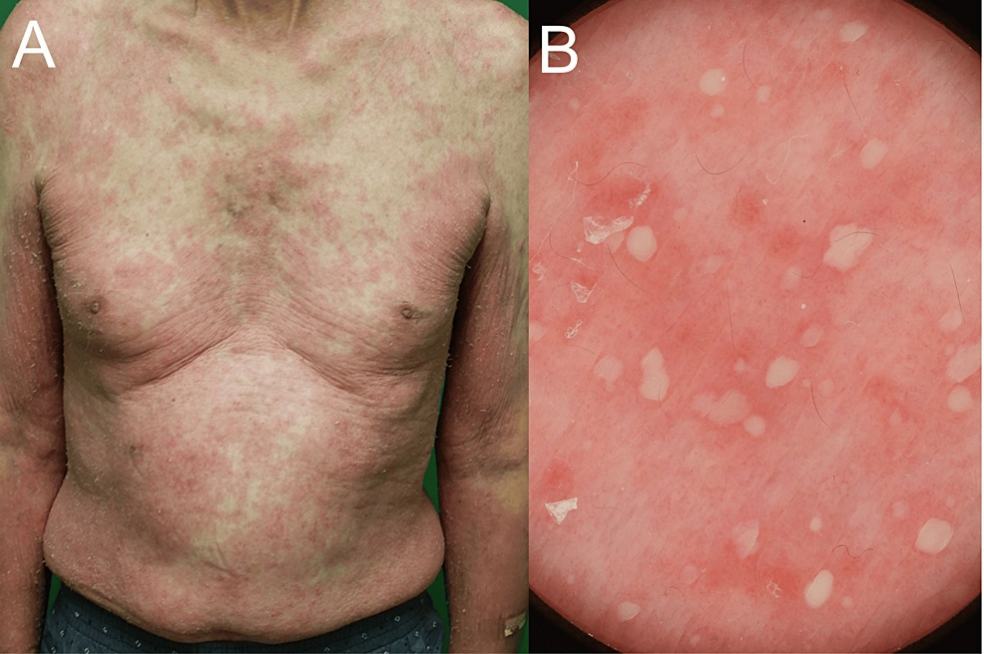
Clinical manifestation of generalized pustular psoriasis (A) Clinical manifestation of pustular psoriasis. Pustules were present on scaly erythematous plaques over the entire body. (B) Dermoscopic findings. Micropustules were distributed across the erythematous plaque.

**Figure 3:**
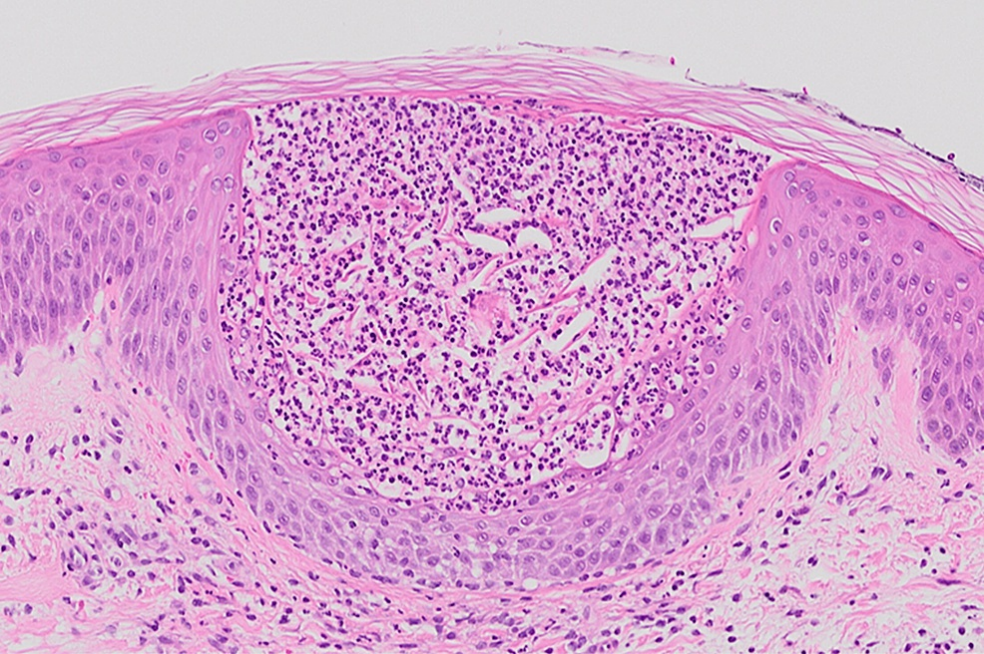
Histological findings Kogoj’s microabscess was observed in the epidermis with clusters of neutrophils, and parakeratosis was present in the stratum corneum.

A 75-year-old male with a decade-long history of managing concomitant psoriasis vulgaris and BP presented with new-onset pustules on erythematous skin on the trunk and limbs (Figure 1A, 1B). He had a fever of 37.5 °C and increased white blood cell counts (14,400/μL) and CRP (12.45 mg/dl). His condition had been stable under a regimen of 20.25 mg oral betamethasone for BP and 200 mg cyclosporine for psoriasis vulgaris. Small pustules were located on the scaly erythematous plaques on the whole body (Figure 2A, 2B). A skin biopsy taken from a pustular plaque revealed prominent spongiform pustules of Kogoj’s microabscess with clusters of neutrophils in the epidermis and parakeratosis in the stratum corneum (Figure 3). Based on the histological examination, we diagnosed his skin eruption as pustular psoriasis. The patient was treated with 900 mg of spesolimab, resulting in a rapid resolution of pustules, although erythema persisted. A subsequent 900 mg dose of spesolimab was administered one week later, leading to complete remission of erythema without exacerbating the bullous lesions. Continuing with 0.25 mg of betamethasone and 200 mg of cyclosporine for BP and psoriasis has maintained disease stability without any recurrence of pustular psoriasis.

## Discussion

The occurrence of pustular lesions in patients with BP and psoriasis undergoing corticosteroid therapy poses a significant challenge, as these treatments can provoke pustular flares in psoriatic skin [6,7]. The complexities of treating such concurrent conditions necessitate a delicate balance to manage one without exacerbating the other [8]. The introduction of spesolimab, an anti-IL-36 receptor antibody, offers a promising therapeutic option by effectively managing pustular psoriasis [9] while mitigating the risk of aggravating BP.

IL-36, a cytokine involved in inflammatory responses, plays a crucial role in various dermatological disorders [10-12]. It is known to induce the production of other pro-inflammatory cytokines and chemokines, recruit immune cells, and amplify inflammatory pathways in the skin. The connection between IL-36 and BP, however, remains underexplored. Interestingly, IL-36 can potentially induce IL-17 production, a cytokine found to be elevated in the skin of BP patients [13]. IL-17 and its associated Th17 cells are known contributors to the inflammation seen in BP, which might suggest a link between IL-36-mediated pathways and BP exacerbation [14].

Evidence supports the effectiveness of IL-17 blockade in managing BP, highlighting the therapeutic potential of targeting IL-17 in cases where IL-36 may indirectly contribute to disease pathology [15,16]. Conversely, treatment with anti-IL-17 antibodies has been linked to the rare development of BP in patients with psoriasis [17]. Furthermore, IL-1 also plays a vital role in GPP pathogenesis [18], suggesting that these pathways are intricately connected and that their modulation can have unpredictable effects on coexisting conditions.

Given these dynamics, the successful management of pustular psoriasis with spesolimab in a patient suffering from concurrent BP is notable. It underscores the potential of IL-36 inhibition not only for controlling pustular psoriasis but also for providing a safer treatment pathway for patients with coexisting autoimmune dermatological conditions. The case encourages further investigation into the specific roles of IL-36 in BP and the broader implications of its inhibition. Such studies are essential for developing more nuanced treatments that can address the complexities of patients with multiple overlapping autoimmune diseases, enhancing both safety and efficacy.

## Conclusions

This case report highlights the successful treatment of a patient with concurrent BP and pustular psoriasis using spesolimab, an anti-IL-36 neutralizing antibody. This underscores the importance of targeted therapies in managing complex autoimmune diseases and suggests a potential role for IL-36 inhibitors in such cases, warranting further research.
